# Supplementary material for: Impact of artificial feeding on the developmental cycle of two triatomine species
Source: PLoS One. 2025 May 12;20(5):e0323090. doi: 10.1371/journal.pone.0323090 (PMC12101860; doi:10.1371/journal.pone.0323090)
Supplement: S1 Text — (DOCX) [file pone.0323090.s001.docx]

**Supplementary material** **text S1**

GEE is advantageous for capturing trends and variations over the study period, accommodating various explanatory variables and treatments, calculating the individual contribution of each variable or treatment to the overall variance, and handling datasets with missing measurements for individual samples at certain time points.
