# Supplementary material for: Impact of artificial feeding on the developmental cycle of two triatomine species
Source: PLoS One. 2025 May 12;20(5):e0323090. doi: 10.1371/journal.pone.0323090 (PMC12101860; doi:10.1371/journal.pone.0323090)
Supplement: S1 Table — (PDF) [file pone.0323090.s002.pdf]

Supplementary Table 1. Average development time (in days) of triatomines fed on chickens, artificial feeder, or alternating feeding methods across different instar stages.

**Instar**	***T. infestans***	***P. megistus***
N* 1	62	76
N 2	171	175
N 3	272	231
N 4	518	291
N 5	413	306
Adult	484	231
**Total**	**1920**	**1310**

N*= Nymph
