# Supplementary figures and images for: Impact of artificial feeding on the developmental cycle of two triatomine species
Source: PLoS One. 2025 May 12;20(5):e0323090. doi: 10.1371/journal.pone.0323090 (PMC12101860; doi:10.1371/journal.pone.0323090)

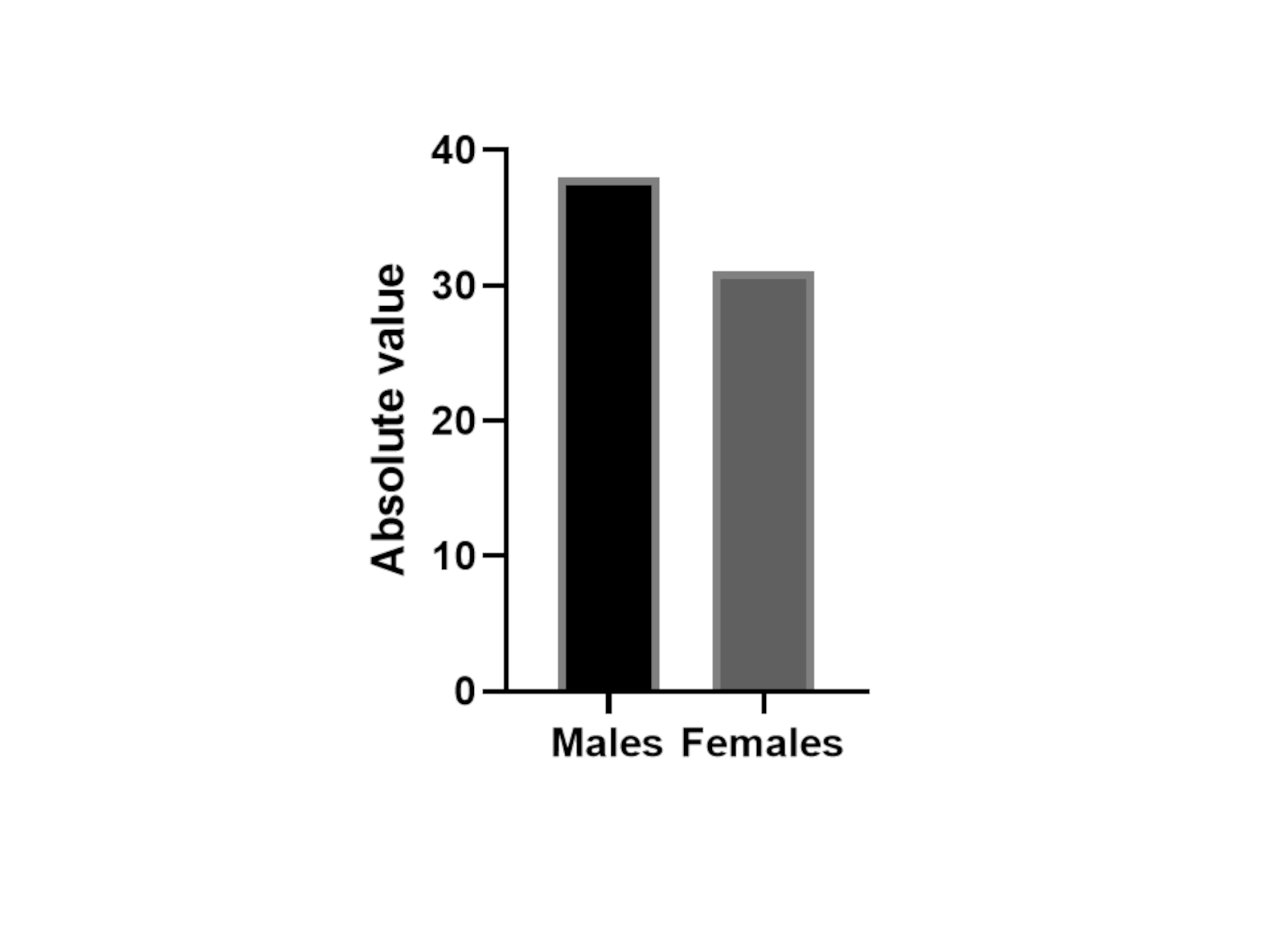

Supplement: S1 Fig — (TIF) [file pone.0323090.s003.tif]

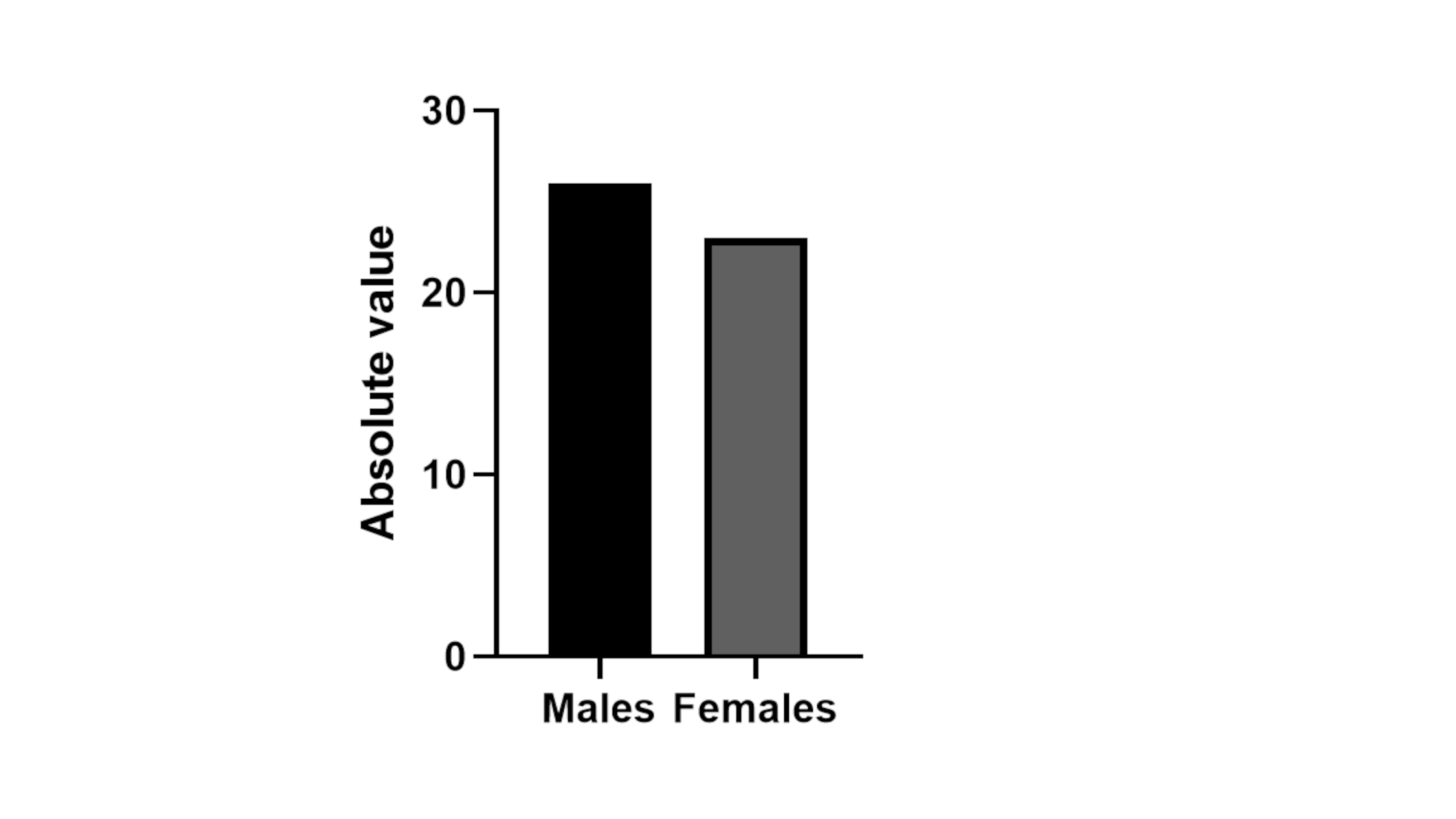

Supplement: S2 Fig — (TIF) [file pone.0323090.s004.tif]
