# Supplementary material for: Impact of artificial feeding on the developmental cycle of two triatomine species
Source: PLoS One. 2025 May 12;20(5):e0323090. doi: 10.1371/journal.pone.0323090 (PMC12101860; doi:10.1371/journal.pone.0323090)
Supplement: S2 Table — Interaction fixed at N1 and the artificial feeder group. (PDF) [file pone.0323090.s005.pdf]

S 2: Table 2: Model of interaction between the different experimental groups and developmental instars when comparing the number of blood feedings required to trigger molting in *P. megistus*. Interaction fixed at N1 and the artificial group.

Description of Interaction	Estimate	p-value	IC* (95%) - Estimate
Alternated	0.212	0.044	[-0.419; -0.005]
Chicken	0.910	< 0.001	[-1.077; -0.744]
N2	0.697	< 0.001	[0.538; 0.857]
N3	1.254	< 0.001	[1.06; 1.448]
N4	1.248	< 0.001	[1.048; 1.449]
N5	1.089	<0.001	[0.86; 1.318]
Alternated	-0.171	0.301	[-0.495; 0.153]
Chicken	0.089	0.541	[-0.377; 0.198]
Alternated	-0.412	0.005	[-0.702; -0.123]
Chicken	-0.378	0.008	[-0.658; -0.098]
Alternated	-0.282	0.078	[-0.595; 0.031]
Chicken	-0.052	0.723	[-0.337; 0.234]
Alternated	0.179	0.299	[-0.158; 0.515]
Chicken	0.572	0.001	[0.243; 0.901]

GEE Poisson model; *CI: confidence interval.
