# Supplementary material for: Impact of artificial feeding on the developmental cycle of two triatomine species
Source: PLoS One. 2025 May 12;20(5):e0323090. doi: 10.1371/journal.pone.0323090 (PMC12101860; doi:10.1371/journal.pone.0323090)
Supplement: S3 Table — (PDF) [file pone.0323090.s006.pdf]

S 3Table 3: Descriptive measures of weight gain by developmental instar of *P. megistus* considering the three feeding methods.

Instar	Minimum	IQ*	Median	Mean	3Q**	Maximum	SD***
**N1**	0	0.0014	0.0028	0.0029	0.0034	0.0202	0.0023
**N2**	7e-04	0.0041	0.0071	0.009	0.0112	0.1031	0.009
**N3**	0.0013	0.0095	0.0163	0.0253	0.0293	0.4899	0.038
**N4**	0.0021	0.0253	0.047	0.0722	0.0938	0.5639	0.0738
**N5**	0.003	0.0737	0.1345	0.1884	0.2834	0.8793	0.163

***IQ: 1st quartile; **3Q: 3rd quartile; ***SD: standard deviation**
