# Supplementary material for: Impact of artificial feeding on the developmental cycle of two triatomine species
Source: PLoS One. 2025 May 12;20(5):e0323090. doi: 10.1371/journal.pone.0323090 (PMC12101860; doi:10.1371/journal.pone.0323090)
Supplement: S4 Table — Interaction between group and stage. The reference instar in all analyses was N1. (PDF) [file pone.0323090.s007.pdf]

S 4 Table 4: Weight gain of N1–N5 nymphs in *P. megistus*. Interaction between group and stage. The reference instar in all analyses was N1.

**Type of Blood Offering**	**Group Interaction**	**Estimate**	**p-value**	**CI (95%) - Estimate**
Artificial feeder	Alternated	-0.001	0.382	[-0.0032; 0.0012]
	Chicken	-0.001	0.063	[-0.0029; 1e-04]
	N2	0.004	< 0.001	[0.0022; 0.0067]
	N3	0.014	< 0.001	[0.01; 0.0175]
	N4	0.033	< 0.001	[0.0205; 0.045]
	N5	0.083	< 0.001	[0.0617; 0.1041]
	Alternated: N2	0.007	0.029	[7e-04; 0.0134]
	Chicken: N2	0.006	0.01	[0.0015; 0.0106]
	Alternated: N3	0.009	0.004	[0.0029; 0.0152]
	Chicken: N3	0.014	< 0.001	[0.0074; 0.0212]
	Alternated: N4	0.018	0.023	[0.0026; 0.0344]
	Chicken: N4	0.045	< 0.001	[0.0238; 0.067]
	Alternated: N5	0.034	0.077	[-0.0037; 0.0726]
	Chicken: N5	0.079	< 0.001	[0.0368; 0.1205]
Alternated	Chicken	0.000	0.649	[-0.0021; 0.0013]
	N2	0.011	< 0.001	[0.0056; 0.0174]
	N3	0.023	< 0.001	[0.0179; 0.0278]
	N4	0.051	< 0.001	[0.041; 0.0614]
	N5	0.117	< 0.001	[0.0856; 0.149]
	Chicken: N2	0.001	0.772	[-0.0081; 0.006]
	Chicken: N3	0.005	0.179	[-0.0024; 0.0128]
	Chicken: N4	0.027	0.01	[0.0064; 0.0474]
	Chicken: N5	0.044	0.072	[-0.0039; 0.0922]
