# Supplementary material for: Impact of artificial feeding on the developmental cycle of two triatomine species
Source: PLoS One. 2025 May 12;20(5):e0323090. doi: 10.1371/journal.pone.0323090 (PMC12101860; doi:10.1371/journal.pone.0323090)
Supplement: S5 Table — (PDF) [file pone.0323090.s008.pdf]

S 5 Table 5: Descriptive measures of weight gain according to sex for *P. megistus* fed on chickens, artificially and alternately.

Sex	Minimum	IQ*	Median	Mean	3Q**	Maximum	SD***
**Male**	1.162	1.23	1.706	1.719	1.908	3.072	0.518
**Female**	0.351	0.817	1.644	1.506	1.947	2.986	0.709

*IQ: 1st Quartile; **3Q: 3rd Quartile; ***SD: Standard Deviation
