# Supplementary material for: Impact of artificial feeding on the developmental cycle of two triatomine species
Source: PLoS One. 2025 May 12;20(5):e0323090. doi: 10.1371/journal.pone.0323090 (PMC12101860; doi:10.1371/journal.pone.0323090)
Supplement: Attachment — Submitted filename: Response to Reviewers.docx [file pone.0323090.s010.docx]

Dear reviewers, we appreciate your suggestions and have accepted almost all of them. Below are the point-by-point responses.

1) Line 56 “Vinchuca” is not the only vernacular Spanish name given to triatomines. “Chinche besucona”, “chinche”, “pito” and also employed, depending on the country. Please, check and complete.

**Authors:** The names were added as suggested by the reviewer

2) Lines 60-61. The feasibility of rearing triatomines in vitro has long been demonstrated. Please rephrase to make it clear that you are interested in large-scale rearing.

**Authors:** Changes were made as suggested.

3) Lines 68-70. Please rephrase, it seems impractical for your needs and under your conditions, but it has been used successfully for decades.

**Authors:** Changes were made as suggested.

4) Lines 169-175. As indicated in several cited references, it is crucial to precise the treatment and conditioning of the blood used for in vitro feeding. Parameters as how long after extraction blood was proposed to bugs, how was it stored in between, the exact composition and amount of anticoagulant solutions, the exact nature and treatment of latex membranes, the type of artificial feeder used, the exact temperature of the blood (and not that of the water bath) are just some of them.

**Authors:** Thank you for your comments. Please note that the information has been added in lines 206-221. The blood used in the artificial feedings was 10% citrated rabbit blood (25g P.A. citrate, 8g citric acid, and 24.5g glucose). This blood is collected, prepared, and sterilized at the Institute of Science and Technology in Biomodels (ICTB)/Fiocruz. It is packaged in an autoclavable glass bottle with a rubber cap, sealed, and distributed chilled on dry ice to the laboratories. In the laboratory, the blood is immediately stored at -20°C and used within 10 days of being collected.

The artificial feeding device used consisted of a glass apparatus, made by a craftsman in the shape of a baby bottle, with an external compartment for circulating water heated by an attached water bath and an internal compartment containing the blood. The water temperature was set at 40°C, and the blood temperature in the bottles remained at 39.5°C. The membrane used to cover the feeding bottle was a latex membrane. These were obtained from natural latex gloves, which were cut into 10x10 cm pieces and washed with Type I water to remove dust. The dried latex membrane was used to seal the mouth of the glass apparatus with the help of an elastic band, simulating the skin of a live animal that was pierced by the insect's mouthparts during feeding (Lazzari, 2014).

5) The authors repeatedly point out that previous reports on in vitro feeding of triatomines do not follow the bugs over several generations, as an argument to justify the need for their study. However, the manuscript only presents what happens along a single generation, so the previous criticism applies to them. My advice would be to provide solid arguments justifying the rationale for a study in 2024 that is not much different from many others done decades ago.

**Authors:** The passage between lines 110 and 113 was moved to lines 131–135. The sentence, 'One of these gaps involves the effectiveness and suitability of rearing insects exclusively with artificial feeders over multiple generations in an insectary,' between lines 115 and 118, was removed from the manuscript. Additionally, the aspect highlighted by the reviewer was revised throughout the manuscript (lines 573–580; 587–591; 594-598;764–776), and minor changes were made to the writing of the last paragraph of the introduction. We hope these adjustments have met your expectations.

6) A major inconsistency that seems unjustified in the current version is why the authors chose to use different species as blood donors and for live host feeding. Why not use the same blood? This would have revealed differences in the methods, whereas in the present experiment the effect of donor and method cannot be distinguished. This reason is probably practical, but needs to be explained.

**Authors:** We understand the reviewer's questions and hope to clarify this point. It is important to note that working with live animals has not been an easy task in the scientific environment, particularly in Brazil. This work is the result of various questions and recommendations from the Ethics Committee on the Use of Animals. The license obtained from CEUA to keep triatomines in an insectary, after a 2.5-year process, does not allow us to breed rabbits at our institution to be used as a source of blood for the triatomines. For this reason, we use the license granted to ICTB/Fiocruz (as mentioned in question 4). ICTB is the technical-scientific unit of Fiocruz responsible for studies, development, and production in Laboratory Animal Science. They prepare and supply blood from other animals, such as rodents and lagomorphs, to other Fiocruz units. Although we have a license to use chickens to provide blood for feeding triatomines kept in the institutional insectary, we lack the expertise and equipment at our institution to prepare the blood, monitor the health of the animals, and carry out pre-clinical analyses on animals bred to ensure quality control of the blood for use in the artificial feeder. Therefore, for practical reasons, the same blood donors were not used in the tests in this study.

7) Some sections of the manuscript are inappropriately long. The detailed description of the statistical treatment of the data is interesting, but can be presented in supplementary material. The results section is also unnecessarily long, but for a different reason: there is redundancy in the information presented in the tables and in the text.

**Authors:** We appreciate your suggestions. Regarding the statistical analyses, we have removed the paragraph describing the GEE test from the manuscript. However, due to the complexity of the analyses and models employed, we believe that the details and description of the criteria used in decision-making should remain in the manuscript.
Regarding the results section, we completely agree with the reviewer and have removed the redundant information.

8) The discussion focuses on the advantages of in vivo over in vitro feeding, which, as the authors say in the introduction, is now well acknowledged. Simply stating that in vitro is not the best and that this strategy might be potentially problematic for mass rearing is much less than most educated readers of PLoS One would expect. In the opinion of this reviewer, this study provides an interesting opportunity to discuss the trade-off that scientists face today, i.e. optimising efforts and resources to advance public health science versus animal welfare regulations. In particular in Chagas endemic countries, where the need of advances is high and the resources usually limited. This problematic goes beyond Brazilian legislation and triatomine rearing.

**Authors:** We appreciate your comments. We have expanded the discussion, addressing the points highlighted by the reviewer. A paragraph was added between lines 573 and 580, and a sentence was added between lines 587-591 and 594-598. Finally, in the last paragraph, lines 774-776 were added, and the ending of this paragraph was rewritten. We hope that these additions are sufficient, as we do not intend to engage in discussions that involve, including, philosophical aspects, which would be more appropriate for another type or format of manuscript.
